# Is there a prognostic value of CMR derived 3D right ventricular geometry and function?

**DOI:** 10.1186/1532-429X-11-S1-P59

**Published:** 2009-01-28

**Authors:** Vikas K Rathi, Vinayak Hegde, Srinivas Murali, Madhavi Akkineni, June A Yamrozik, Ronald B Williams, Geetha Rayarao, Diane A Vido, Mark Doyle, Robert WW Biederman

**Affiliations:** Allegheny General Hospital, The Gerald McGinnis Cardiovascular Institute, Pittsburgh, PA USA

**Keywords:** Pulmonary Hypertension, Right Ventricular, Pulmonary Arterial Hypertension, Idiopathic Pulmonary Arterial Hypertension, Relative Wall Thickness

## Introduction

Pulmonary hypertension (PH) accounts for substantial morbidity and mortality. Advances in cardiac imaging have enabled the 3 Dimensional (3D) study of right ventricular (RV) geometry and functional parameters; however, their impact on patient outcomes in various groups of patients (pts) such as idiopathic pulmonary arterial hypertension (IPAH, WHO Group I) when compared to PH secondary to other causes (SPH, WHO Group II–V) has not been fully studied.

## Aim

We propose that the RV volume and geometry in IPAH and SPH pt groups will correlate with a clinically important prognostic parameter such as 6-minute walk distance (6 MWD).

## Methods

Pts (n = 32) with severe PH were divided into 2 groups: 15 pts with IPAH and 17 pts with SPH. All pts underwent cardiac MRI (CMR) (1.5 T GE, Milwaukee, WI) and measurements of 3D RV end-diastolic and end-systolic volumes (EDV, ESV), ejection fraction (EF), mass and relative wall thickness (RWT), all indexed to BSA and were compared to 6 MWD.

## Results

There were no differences in the RV geometry or function between the two groups. The mean RV mass index, RWT and RVEF were 34.8 ± 29, 0.19 ± 0.11 and 34 ± 14% in the IPAH group vs 27 ± 10, 0.20 ± 0.11 and 44 ± 15%, in the SPAH group (p = ns). The RWT and RV mass index demonstrated that the RV was eccentrically hypertrophied in both groups despite severe PH. The IPAH pts had slightly higher PA systolic pressure when compared to SPH (84 ± 16 vs 64 ± 24 mmHg, p = 0.04) but had lower pulmonary wedge pressures (10 ± 7 vs. 17 ± 8 mmHg; p < 0.05). The mean 6 MWD was similar between the IPAH vs. SPH group (344 ± 88 vs. 302 ± 98 meters, p = NS). The RVEDVI, RVESVI and RVMI positively correlated with 6 MWD (R = 0.52, 0.48 and 0.50, p < 0.05) of IPAH patients but did not demonstrate significant correlation for SPH pts. Furthermore, the 3D RVEF, 2D TAPSE and ratio of RVMI to RV volume did not demonstrate correlation with the 6 MWD.

## Conclusion

Although IPAH and SPH patients have very similar RV geometry, structure and function, noninvasive CMR metrics only predict 6 MWD for IPAH patients. Clinically important predictors of 6 MWD by CMR metrics for the SPH group, a potentially more heterogeneous group, is not beneficial. These observations highlight the key pathophysiologic differences in the compensatory response of the RV to vastly different pathophysiology despite a more homogeneous response at the level of the RV chamber. This finding may account for the observed differences in treatment response and clinical outcomes in these patients.

